# GIS-based assessment of photovoltaic solar potential on building rooftops in equatorial urban areas

**DOI:** 10.1016/j.heliyon.2024.e41425

**Published:** 2024-12-21

**Authors:** Andrés Idrovo-Macancela, Marco Velecela-Zhindón, Antonio Barragán-Escandón, Esteban Zalamea-León, Danilo Mejía-Coronel

**Affiliations:** a Energy Transition Research Group (GITE), Universidad Politécnica Salesiana, 010103, Cuenca, Ecuador; b Universidad de Cuenca, Facultad de Arquitectura y Urbanismo, Av 12 de abril y Agustin Cueva, Cuenca, Ecuador; c Universidad de Cuenca, Laboratorio de Ecología Acuática (LEA), Balzay Campus, Cuenca, 010107, Ecuador

**Keywords:** Solar rooftops, LiDAR technology, Urban planning, Urban energy self-sufficiency

## Abstract

Installing photovoltaic systems (PVs) on building rooftops is a viable and sustainable alternative to meet the growing demand for electricity in cities. This work develops a methodology that uses LiDAR (laser imaging detection and ranging) technology and roof footprints to obtain a three-dimensional representation of the rooftops in the urban centre of Santa Isabel (Azuay, Ecuador). This allowed the determination of characteristics such as area, slope, orientation, and received solar radiation, making it possible to calculate the rooftop's theoretical, technical, and economic photovoltaic potential. It was found that 68.8 % of the total roof surface is suitable for PV capture, with which a theoretical photovoltaic potential of 62.39 GWh can be achieved. The annual technical photovoltaic potential using silicon panels was calculated at 4.85 GWh, which could supply 4.97 times the demand of the analyzed neighbourhood. To determine the economic potential, the peak power of a solar project was calculated to match the electricity demand of the analysis area, with a levelized cost of energy of 12.37 c$/kWh. This project could prevent the emission of 6805 tons of CO₂ into the environment during its useful life. The methodology developed in this study can be replicated in other areas to determine their photovoltaic potential and contribute to the diversification and decentralization of electricity generation systems, as well as transform Ecuador's energy matrix.

## Highlights

• The developed method is useful for promoting urban self-energy supply.
•LIDAR facilitates determining photovoltaic solar potential in urban environments.
•The proposed method is replicable to other urban areas.
•In urban planning, photovoltaic solar potential should be included.

## Acronyms

DSM: Digital Surface Model
GHG: Greenhouse Gases
GIS: Geographic Information System
GHI: Global Horizontal Irradiation
IRENA: International Renewable Energy Agency
LiDAR: Laser Imaging Detection and Ranging
LOSPEE: Organic Law of Public Electricity Service
LCOE: Levelized Cost of Energy
NREL: National Renewable Energy Laboratory
PVpot: Photovoltaic Potential
PVs: Photovoltaic systems
SR: Solar Radiation

## 1 Introduction

Currently, most of the population is concentrated in urban areas, accounting for more than 70 % of global energy consumption [1]. According to the UN, energy production is responsible for 60 % of global greenhouse gas (GHG) emissions [2]. Projections indicate that by 2050, 68 % of the world's population will live in urban areas [3], which is driving initiatives for urban environments to incorporate energy generation systems that utilize local resources, reducing dependence on hydrocarbons and preventing the degradation of wilderness areas and ecosystems. Promoting the use of integrated PVs in cities enhances resilience and potentially reduces urban pollution. Therefore, determining the photovoltaic potential (PVpot) in cities allows for assessing the theoretical capacity for possible urban self-sufficiency.

The installation of PV systems in buildings is an innovative and sustainable solution to current energy challenges, offering multiple benefits across various areas. In terms of energy efficiency, it enables on-site electricity production with high cost-effectiveness [4]. Regarding environmental impact, PVs reduce dependence on fossil fuels and consequently decrease greenhouse gas (GHG) emissions [5]. From architectural and technological flexibility perspectives, these systems integrate aesthetically, scalably, and functionally into structures through the use of photovoltaic tiles, roofs, or windows [6,7].

The National Renewable Energy Laboratory (NREL) has defined the following types of photovoltaic sub-potentials.

• Theoretical, which is determined solely based on solar irradiation measurements specific to each area.
• Technical, calculated based on the performance of PVs and topographical restrictions.
• Economic, which analyzes costs and return rates during the useful life of PVs.
• Market, which studies the feasibility of PVs based on policies, regulations, or investment opportunities [8].

Around 71 % of the world's population lives in countries with a technical PVpot that has a favourable average range between 3.54 and 5 kWh/kWp [9]. In this context, PVs are emerging as a viable option for the electrification and sustainable development of cities. This is because their operation has minimal environmental impact, and they can be easily installed on rooftops and/or building facades [10,11]. By 2023, electricity production through PVs accounted for approximately 5.5 % of the global total [12]. In the same year, in Ecuador, the contribution of these systems (mostly solar power plants) represented only 0.11 % of the total energy produced. The largest percentage came from hydropower plants at 71.68 %, followed by thermal power plants at 26.6 % [13]. The current deployment of PVs in Ecuador contrasts with its significant theoretical PVpot. Its location on the equator and low seasonality provides it with a relatively stable Global Horizontal Irradiation (GHI) index, ranging from 2.9 to 6.3 kWh/m^2^ per day [14]. Additionally, according to Cevallos and Ramos [15], the renewable technology with the greatest capacity to be distributed across the territory is PV. In this sense, this generation is complementary to hydropower generation, while during dry seasons that reduce hydropower capacity, PVs show high generation [16].

The use of distributed generation can be increased through initiatives outlined in the Ecuadorian Constitution (articles 15 and 413) [17], the Organic Law of Energy Competitiveness [18], and regulations regarding distributed generation for the self-supply of regulated electricity consumers (Regulation No. ARCERNNR-008/23) [19]. These initiatives promote the use of renewable energy sources through fiscal and financial incentives such as tax and VAT exemptions, reduced tax burdens, streamlined procedures, preferential energy dispatch, and more. Another key factor for the adoption of PVs is that they have become the most competitive renewable technology. According to the International Renewable Energy Agency (IRENA), in the last decade, their levelized cost of energy (LCOE) has decreased by 88 %. During the same period, the prices of silicon solar panels dropped by 88–95 %, and this trend is expected to continue in the future [20].

According to the 2023 Ecuadorian electricity sector statistics [13], 869 PV projects for self-supply have been authorized nationwide, reaching an installed capacity of 38.37 MW. Locally, the electric utility Centrosur reports the operation of 73 of these projects with a total capacity of 622.44 kW. The mentioned projects used silicon panels due to their high efficiency and durability. However, for large-scale implementation, it is essential to consider emerging technologies such as thin-film PV. Although their efficiency is lower, their lightweight and flexible design allows for easy integration on various types of surfaces [21,22].

In 2023, 120 MW were awarded for large-scale photovoltaic power plants, while in 2024, 1580 MW were identified across seven PV projects in a preliminary assessment. Although the solar potential is significant, there is a slow pace in the implementation of these systems, despite having a supportive legal framework. This can be attributed to the initial investment costs or the lack of accessible information for the public, such as maps of suitable rooftop areas for installing PVs and local policies or support from local governments, which have been crucial in areas where PV self-supply has become widespread.

To promote the development of solar cities in Ecuador, it is essential to start with the development of methodologies to determine PVpot. These methodologies provide useful information that can influence a consumer's decision to implement PVs for self-supply. This is essential as a preliminary step to support policies that ensure the implementation of technologies that harness solar energy as a power source [23].

After extensive research, we have identified that in Ecuador, processes for determining PV potential (PVpot) in large urban environments have not been significantly developed. Consequently, the development and application of methodologies related to this process remain in an initial phase. To address this limitation, this study presents, implements, and evaluates a replicable methodology, most of which procedures are automated. This approach will allow urban planners to identify PVpot in large urban areas using advanced technologies such as LiDAR, thereby facilitating the integration of renewable energy as a key element in local sustainability planning.

The structure of this article is organized as follows: Section 2 presents a review of studies that have calculated PVpot in urban environments. Section 3 describes the study area, while Section 4 outlines the applied methodology. The results, discussion, and conclusions are presented in Sections 5, 6, 7, respectively.

## 2 Literature review

An effective study to determine the viability of implementing PVs in urban structures should adhere to the following principles: estimate the total roof area, calculate the area suitable for PV installation, determine the incident solar radiation (SR) on the roof, evaluate the technical PVpot, and, as an additional measure of effectiveness, calculate the economic PVpot [24].

Melius et al. [25] hade defined four methodological branches for roof analysis that align with these principles.

• *Constant Value Methods:* Used in studies [[26], [27], [28]]. These methods allow for a quick estimation but lack precision as they assume various geometric characteristics of the roofs.
• *Manual Methods:* Referenced in Refs. [[29], [30], [31]]. This approach uses, for example, aerial images and/or on-site inspections to manually select roofs with the best PVPot. While precise, these methods are time-consuming and challenging to replicate.
• *GIS Methods:* These analyze the 3D model of an area generated with technologies such as orthophotos or LiDAR [[32], [33], [34]]. They offer high replicability but require significant computational resources and time.

### 2.1 Reference cases

An accurate assessment of PVPot in cities is essential for efficiently harnessing this renewable energy source in urban environments. In this context, the combined use of LiDAR (Light Detection and Ranging) data for PVs has emerged as a valuable approach to achieving this goal. LiDAR permitted the determination of variables such as incident SR, surface, orientation and slope, which are key for installing PVs, thereby creating significant synergy between both technologies. However, the extent of the analyzed area and the variability in roof shapes present a challenge for scaling up the use of LiDAR in determining PVPot, as this process directly depends on the available computing capabilities.

LiDAR sensors operate by sending laser pulses and measuring the time and speed of their return when reflected by objects. This data has been used to determine the distance and shape of the analyzed object. Each data point had been geographically referenced by a highly precise satellite positioning system and an inertial measurement unit incorporated into the capture system [35]. The most commonly used sensor is the mechanical LiDAR, which rotates horizontally in a set of vertical configurations, allowing it to calculate in 360° a certain number of points or the position of objects, enabling the establishment of their shape and dimensions [36].

D. Lingfors et al. [37], highlighted that the resolutions used by LiDAR sensors limit the accuracy of the results; however, with approximation routines, it is possible to achieve good precision even with low resolutions for photovoltaic estimation. Although high resolution offers greater accuracy, low resolutions are satisfactory for evaluating roof types in areas with a homogeneous distribution of buildings. In this case, high-resolution LiDAR is crucial for assessing the orientation of PVs, although potential discrepancies in finding azimuths from aerial images are noted.

In Leeds (United Kingdom), LiDAR was applied and demonstrated that compared to using aerial photography, LiDAR achieves an efficiency of 81 % in small buildings, and its use is feasible in larger areas in less time than aerial photography [38]. The study by Srećković et al. [39] proposes an innovative procedure to determine the most suitable roof surfaces for installing PVs in urban environments. They assess PV capacity based on LiDAR data and pyranometer measurements, considering generation and load profiles to minimize annual grid losses.

In Lisbon (Portugal), using LiDAR sensors, 538 buildings were identified with an annual generation of 11.5 GWh (7 MW of power) from the installation of PVs, which could supply 48 % of the energy demand [40]. This study highlights that for high PV penetration, the presence of horizontal surfaces on buildings provides accurate PVpot estimates, avoiding complex shading analyses. Recent research by Brito et al. [41] emphasizes the relevance of PVpot in facades and other vertical features. The annual analysis reveals that the potential in roofs and facades can exceed local demand, contributing up to 75 % of the total demand. Including facade radiation is essential to meet peak demand in winter.

In Istanbul (Turkey), this technology was also applied over an area of 5400 km^2^, covering 39 districts with 1.3 million buildings, and an estimated annual potential of 30.8 TWh was defined, representing 67 % of the total electricity demand [42]. In this case, it was determined that climatic variability and the presence of shadows are significant influencing factors in PV energy production. To reach this conclusion, an integrated approach combining LiDAR data with meteorological measurements was applied to validate electricity production models. In Apeldoorn (Netherlands), the PVpot is established at 319.9 Wp for residential buildings, equivalent to 283.94 GWh annually, and compared to the residential energy demand of 230 GWh, it could cover 100 % of the required electricity [43].

Redweik et al. [35] used LiDAR to generate a digital surface model (DSM) of the University of Lisbon campus. This work conducted a decade ago, demonstrated the potential of LiDAR, allowing for the estimation of the theoretical PVPot of roofs (34 GWh/year) and facades (19 GWh/year). Bayrakci Boz et al. [44] proposed a method combining LiDAR and building footprints to identify suitable areas for PV installation in an urban area of Philadelphia, USA. Roofs were analyzed based on orientation, slope, shading, and available area, concluding that 48.6 % of roof areas are suitable for PVs.

Lukač et al. [45] presented a LiDAR procedure combined with historical radiation measurements, also considering inverter degradation and non-linear efficiency, as well as different types of panels. This study analyzed the roofs of 0.5 km^2^ in Maribor, Slovenia, concluding that the maximum PVpot (530 Wh/m^2^ per day) would be achieved with monocrystalline panels. In Ludwigsburg, Germany, SimStadt was used to analyze 3D building models created from LiDAR data and stereo photographs. Radiation reduction factors on roofs were determined, estimating that the technical PVPot could supply 77 % of the region electricity consumption. Considering the economic PVPot feasibility, the supply would be a little lower, reaching 56 % [46].

Mansouri et al. [47] used a methodological approach similar to that proposed by Bayrakci Boz et al. [44] to determine the technical and economic PVpot of rooftops in Alberta, Canada. The technical PVpot would cover 38 % of the 2016 demand, while the economic PVpot concludes that 96 % of these photovoltaic rooftops would be profitable. The study [48] conducts a neighbourhood-level analysis of theoretical PVpot in Auckland, New Zealand. Lastools and Python are used to process LiDAR data, while ArcGIS calculates the incident SR on rooftops. The results identify neighbourhoods with the highest PVpot that could benefit from policy support for the implementation of PVs.

In [49], rooftops were modeled using aerial images and LiDAR, and then the PVPot was estimated with a photovoltaic module adjustment algorithm. The algorithm placed 17.5 % fewer PVs compared to actual panel data installed in Eindhoven, Netherlands. It is concluded that the calculated performance of PVs with rapid scanning is more accurate when using software like Solar Monkey, which considers the horizon profile. Brito et al. present a 3D model of the PVPot for two urban centers in Lisbon, Portugal [50]. Using LiDAR, the SOL algorithm implemented in Matlab, and considering anisotropic diffuse irradiation, they calculate the theoretical PVPot for rooftops and facades as 2531.93 and 684.37 kWh/m^2^, respectively.

Jiménez et al. [51] applied a LiDAR methodology complemented with orthophotos to determine the geometry of the roofs in 24 km^2^ of Ávila, Spain. This analysis is validated as the results do not differ from the characteristics of the roofs extracted from a more detailed LiDAR point cloud. The authors determine that 56 % of the identified roofs are suitable for the installation of PVs. In Ref. [52], the annual energy production of photovoltaic roofs is estimated in Vaihingen (2670 MWh), Pekre (451 MWh), and New York (2541 MWh). The authors consider the geometric characteristics of the roofs to apply a method for the optimal placement of large-scale PVs to maximize the PVPot of these areas.

Jurasz et al. [53] also introduced LiDAR to analyze the PVPot of roofs in Wrocław, Poland, examining aspects of electricity demand and environmental factors. The degree of energy self-sufficiency with PVs in various areas of the study area was determined, and a 30 % reduction in emissions related to electricity use was calculated. The PVs installed on the rooftops would have an approximate maximum installed capacity of 850 MWp, which could cover 36 % of the demand.

Gagnon et al. [54] evaluated the technical PVpot of roofs across the U.S. on a national level. The authors use LiDAR and building footprints to determine the suitability of roofs for PV installation based on their characteristics. A statistical model is developed to estimate an annual generation potential of 1432 TWh/year. An interesting study is the one proposed by Montealegre et al. [55] conducted in Zaragoza, Spain. This work determines the capacity of roofs to produce energy (60.9 kWh/m^2^), grow food, and capture rainwater. The rooftops are modeled with LiDAR, and the suitability of these areas is analyzed using a multicriteria decision-making technique.

In the case of Ecuador, Tian et al. [56], determined the technical PVPot of roofs in two urban centers in the Galápagos Islands. The authors use building footprint data, assuming that all roofs are flat for the implementation of PVs. The results indicate that the electrical demand of these areas could be met with PVs installed in an area equivalent to 21 % and 27 % of the total roof surface analyzed, which highlights the outstanding conditions for meeting and exceeding building demands in the excellent Andean equatorial climatic conditions.

In the same region as the current research, Zalamea et al. [57] already used GIS methodology to determine the technical PVPot of rooftops in the central area of Cuenca city, located in the Andean equatorial climate. The authors conclude that with PV installation, up to 148 % of the total urban demand could be covered; however, when considering architectural aspects and implementing PV tiles, this percentage would be reduced to 61 %, based on the efficiency of PV products available in 2016. In another study in this same region [58] an estimate is made of the available roof areas across the entire urban area of Cuenca city. Using cadastral data and calculating reduction factors extrapolated from other urban environments, the technical PVPot is quantified. The results indicate that energy from rooftop PV installations could reach a value of 3.19 times the 2015 electricity demand.

Several previous studies have calculated the PVpot in small urban areas such as residential or commercial centers [59,60], and [61]. All these studies converge on the same conclusion: Ecuador has excellent PVPot, often with the capacity to exceed the electricity demands of the buildings themselves. PVs can sustainably supply a high percentage of the electricity demand in cities, considering that this technology currently meets the three fundamental conditions: environmentally friendly, socially and culturally integrable, and necessary and profitable. Table 1 presents a summary of the reviewed methodologies and results. The summary confirms that, among the different analysis approaches, GIS methods provide the most accurate results [62]. For this reason, LiDAR techniques for estimating rooftop PVPot have become one of the most widely used tools.

**Table 1:** Estimation results of PVPot in urban environments.

Reference	Place/Country	Software/Algorithm	Input data	PVPot surface	Metodology	Main results
[56]	Ecuador		Roof Surface footprint	0.71 km^2^ roof surface	The necessary building footprint area is calculated to meet the demand by implementing PV.	21 % and 27 % of the total roof area analyzed are required to meet the demand for two islands.
[57]	Ecuador	GIS tools BIM SAM PV	Satellital image Urban energy consumption	1.07 km^2^ total urban area	Manual roof characterization using GIS tools.	With the installation of PV systems, up to 148 % of the demand could be covered, whereas if photovoltaic tiles are used, this percentage would decrease to 61 %.
[58]	Ecuador		Cadastre	16.56 km^2^ total urban area	Cadastre data and the calculation of reduction factors are used to determine the technical PVPot.	The PVPot could cover 3.19 times the electricity demand for the year 2015.
[35]	Lisbon-Portugal	SOL algorithm	LiDAR image	0.16 km^2^ total urban area	An algorithm had been used to determine the shadow map and sky view factor. - Direct and diffuse radiation is calculated for the ground, roofs, and facades.	PVPot of roofs: 34 GWh per year. PVPot of facades: 19 GWh per year.
[44]	Philadelphia-USA	ArcGIS	LiDAR image Roof Surface footprint	62.50 km^2^ roof surface	It was detected the geometrical characteristics of roofs to find the PVPot	48.6 % of the roof area is suitable for PV. Technical PVPot: 800 MW.
[45]	Maribor- Slovenia		LiDAR Historical radiation measurements	0.5 km^2^ total urban area	It was estimated the PVPot considering degradation, non-linear inverter efficiency, and different types of solar panels.	The maximum PVPot (530 Wh/m^2^ per day) would be achieved with monocrystalline panels.
[46]	Ludwigsburg - Germany	SimStadt	CityGML 3D building models created by LIDAR	700 km^2^ total urban area	Analysis of 3D models considering the utilization factor of the available area was performed, also calculating the constraints that reduce the available area.	The technical PVPot could cover 77 % of the demand, while the economic PVPot would cover 56 %.
[47]	Lethbridge-Canada	ArcGIS	LiDAR Roof Surface footprint	124.3 km^2^ total urban area	Determination of roof geometric characteristics to calculate the PVPot.	The technical PVPot would cover 38 % of the demand for the year 2016. The economic PVPot concludes that 96 % of these PV roofs would be profitable.
[48]	Auckland - New Zealand	ArcGIS Lastools SolarView	LiDAR Roof Surface footprint	649,141 roofs	The PVPot of several neighborhoods is estimated considering the area occupied by PV on roofs. The results obtained from the proposed solar radiation model are compared with those obtained using SolarView.	The results identify neighborhoods with the highest PVPot that could benefit from policy support for the implementation of PV.
[49]	Eindhoven - Netherlands	- PVGIS - SolarMonkey - PVMD	LiDAR Aerial images	− 145 roofs − 215 roof segments	An algorithm development was performed for adjusting PV modules on roofs. Results from the algorithm were compared to real data accordingly to the number of panels and performance of installed PV in the analysis area.	The algorithm placed 17.5 % fewer panels. The performance of PV Systems calculated with quick scanning is more accurately approximated when using software like Solar Monkey, which takes into account the horizon profile.

The previous cases are methodologies that allow the establishment of urban PVPot. However, it is necessary to refine the results based on architectural utility considerations that automated systems sometimes cannot accurately define. In other words, it is essential to consider useable roofs for alternative uses, the presence of accessory elements such as antennas, lightning rods, or chimneys, the stability and structural material suitability of the roofs, or even accessibility to the roofs for installation and maintenance. This work develops a replicable methodology suitable for equatorial conditions where roof orientations and inclinations are not limiting factors due to the high solar path, situation existing in the equatorial region. The novelty lies in the automation of sizing processes applicable to this latitude, which allows for the exclusion of shaded roof areas with reduced levels of irradiation incidence.

## 3 Research area

**Fig. 1:**
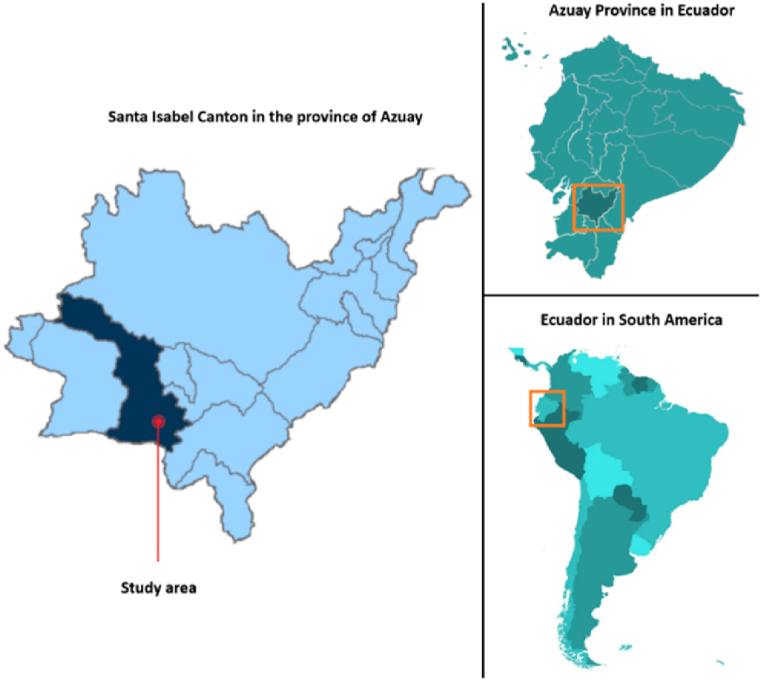
Geographic location of the canton Santa Isabel.

This study was conducted in Santa Isabel, a canton belonging to the province of Azuay, Ecuador. It is located at the coordinates 3°16′27.01″S, 79°18′56.16″W (Fig. 1). The climate of the study area is characterized as dry mesothermal equatorial with average temperatures ranging between 20.9 and 22.6 °C [63]. According to the 2010 census, the population of Santa Isabel was 18,393 inhabitants, and it is projected that by 2030 this population will increase to 33,800 [64]. Santa Isabel is a town located in an Andes Mountain valley at an elevation of 1620 m above sea level in its urban area. It is situated in Climate Zone 2, or the -Humid Warm Zone-according to the Ecuadorian Construction Code, Chapter on Energy Efficiency as the valley conditions moderate the temperature despite the altitude [65]. The average temperature is 19 °C; however, nights are cool, which helps reduce overheating in buildings. Due to its proximity to the equator, seasonal climate variations are minimal throughout the year.

**Fig. 2:**
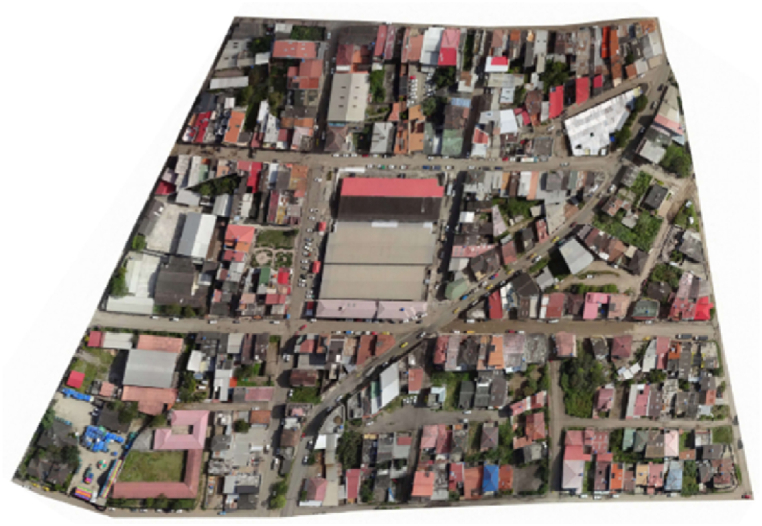
Study area, urban center of Santa Isabel.

According to the research [66], the urban centre of Santa Isabel is located in a region with an average annual GHI of 5 kWh/m^2^ per day. So far, no studies have been reported that estimate the PVpot of this area. Therefore, as a starting point, this research will determine the PVpot at the neighbourhood level of the roofs in this area. A 0.1 km^2^ area belonging to the cantonal centre has been selected (Fig. 2). This area includes residential, commercial, educational, and recreational zones.

## 4 Methodology

In urban environments, artificial and natural elements converge, influencing the amount of solar energy incident on roofs [67]. Determining the suitability of buildings for the implementation of PV systems involves modelling the environment, solar SR, and the available irradiated area [68]. From both functional and aesthetic perspectives, roofs are the most appropriate location for integrating PVs. In regions near the equator, the inclination, orientation, and reduced shading enhances better solar irradiation capture compared to facades [35,69].

An accurate estimation of the PVpot can be achieved through LiDAR technology, which allows for volumetric modelling of a specific location. A 3D representation and its geolocation enable the simulation of available surfaces, shading between buildings, and surrounding elements, while also considering roof inclinations and orientations [70].In this study, the technical PVpot of rooftops in a typical urban center in Ecuador is estimated. A methodology using geographic information systems (GIS) based on data captured by a drone-borne LiDAR sensor is applied.

**Fig. 3:**
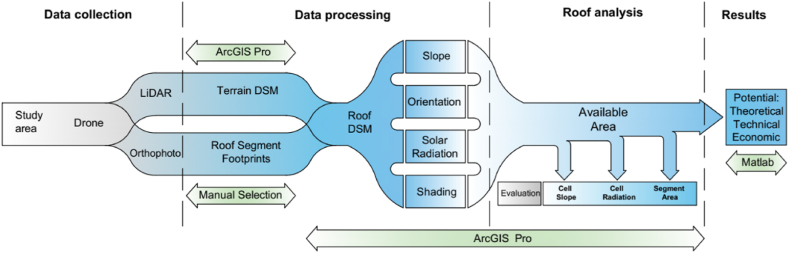
Diagram of the proposed methodology.

The methodology used is shown in Fig. 3, specifying the procedure and software tools utilized. The use of ArcGIS Pro 3.0.2 is considered in conjunction with LiDAR data and a roof footprint layer that delimits each roof in 2D, which was manually created to determine the geometric characteristics of the roofs along with the LiDAR data and exclude areas not identified as roofs. The theoretical, technical, and economic PVpot were calculated using a script developed in Matlab.

This proposal is a replicable method to calculate the theoretical, technical, and economic PVpot of rooftops in an urban area of any size. Using LiDAR, along with roof footprints and the application of reduction factors, it is possible to determine and generate maps of the roof surfaces suitable for installing PVs.

### 4.1 Data collection

The LiDAR data was collected with a LiDAR it Explorer R sensor carried by a drone at an altitude of 100 m. At this height, the instrument's precision is 3.5 cm, and its point density is 71 points/m^2^. The data collection process provides a point cloud in LAS 1.2 format, which can be used to create a DSM of the study area.

**Fig. 4:**
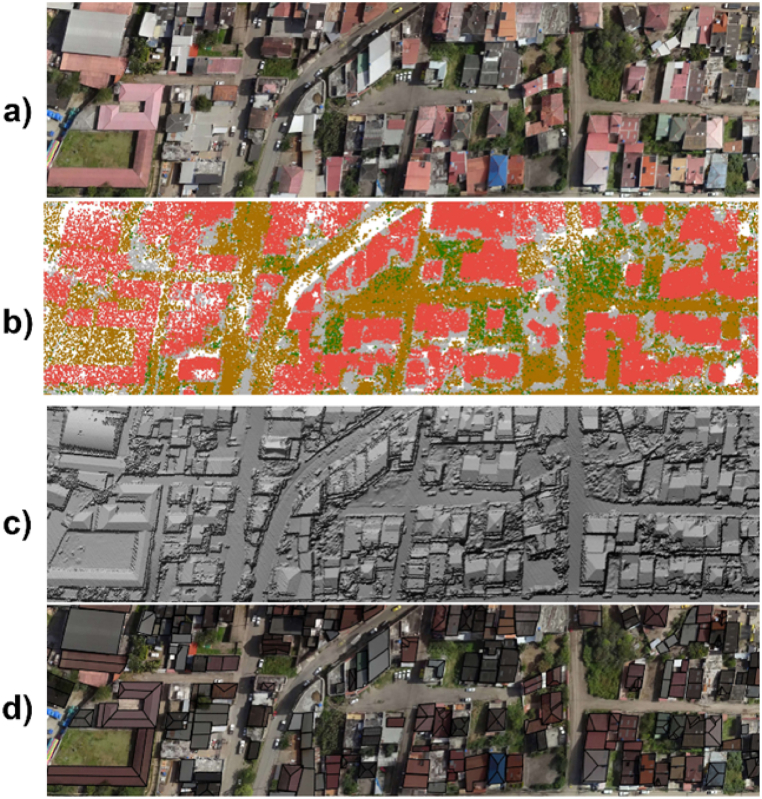
Data from: a) Zenmuse P1 Camera b) LiDAR System; Layers: c) DSM, d) Roof Segments.

Additionally, an orthophoto was obtained using a Zenmuse P1 camera (Fig. 4a), in which each pixel represents an area of 0.06x0.06 m^2^. This orthophoto provides a 2D visual reference to determine the shape of roof segments. Both the LiDAR data and the orthophoto use the Datum WGS 1984/UTM Zone 17S projected coordinate system. This ensures consistency throughout the analysis, as the mentioned data adhere to the same georeferencing system.

### 4.2 Data processing

The LAS file obtained from the analyzed area after the data collection contains a cloud of approximately 19.8 million points categorized by the LiDAR system based on the element that reflected the laser pulse. In ArcGIS, the visualization of this file was configured with that classification to differentiate between ground, buildings, and vegetation (Fig. 4b). To apply the proposed analysis, it is necessary to create the DSM of the neighbourhood (Fig. 4c). This DSM was obtained using the "LAS dataset to raster" tool in ArcGIS [71], configuring it to interpolate using the maximum value method of the point cloud elevations to obtain the heights for the DSM. The result is a 2D cell matrix, known as a raster, where each cell represents an elevation value and covers an area of 0.4 x 0.4 m^2^.

The second spatial layer contains 868 two-dimensional roof segment footprints (Fig. 4d). A manual methodology was applied by creating polygonal entities in ArcGIS to draw these footprints with the help of the orthophoto. A LiDAR method was tested to obtain the footprints, but significant inaccuracies were found since point clouds typically present noise at roof edges [44]. Outdoor terraces have been excluded from this analysis since installing PVs would compromise their function as useable outdoor or recreational spaces.

#### 4.2.1 Roof slope

The production of PVs is affected by the angle of incidence of sunlight on the panels, with greater generation occurring when the rays strike perpendicularly [55]. To accurately estimate the PVpot the slope of the roofs is determined, as PVs are typically installed coplanar with the surfaces of sloped roofs. The inclination of each roof segment was established using the "slope" tool from the Spatial Analyst toolbox in ArcGIS. This tool calculates the rate of change in elevation of the DSM cells relative to their 8 neighbors [72]. At the end of the calculation process, a raster is produced where each cell contains a slope value in degrees, ranging from 0° (horizontal) to 90° (vertical).

**Fig. 5:**
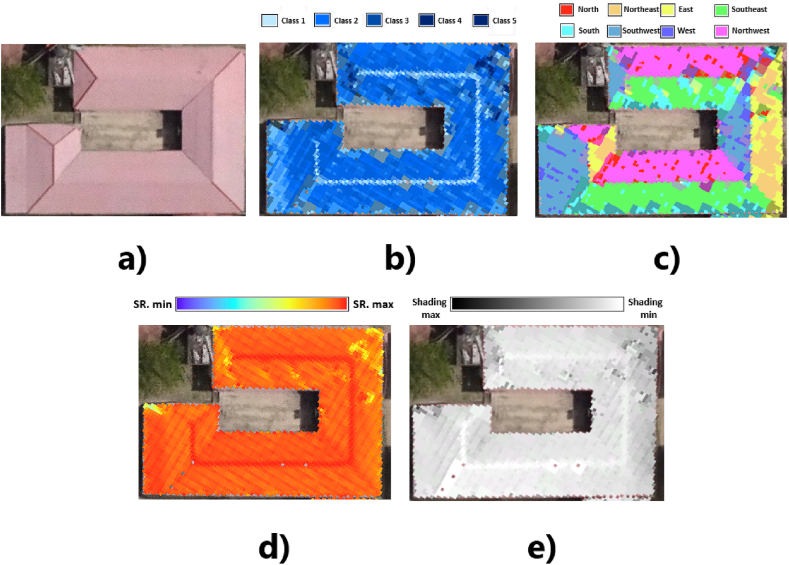
Roof Feature Processing: a) Analyzed roof b) Slope c) Orientation d) Annual Solar Radiation e) Shading.

LiDAR data incorporates noise that introduces variations in slope within the same roof segment with a characteristic inclination [44]. To reduce these variations, the "majority filter" tool in ArcGIS was used, which replaces the value of a cell based on the data from its 8 contiguous cells. The resulting raster reflects the slope of the roof segments as seen in Fig. 5b.

Thus, the cells were classified based on their slope as specified in Table 2. This classification was adapted from Bayrakci Boz et al. [44] to simplify data analysis and management while maintaining higher precision than a basic categorization of roofs as either flat or sloped. This decision is further supported by the consideration that a 5° increase in tilt angle reduces PV efficiency [73]. In Fig. 5(a and b,c and d), roof pixels are presented in colour in concordance with its slope, orientation, shading and irradiance incidence, dependent on the three previous parameters.

**Table 2:** Roof segment slope classes.

Slope value (°)	Class
0–10	1
10–20	2
20–30	3
30–40	4
40–90	5

#### 4.2.2 Orientation

Orientation is another factor that determines the performance of PVs. This variable is defined by the azimuthal angle of the panels (horizontal orientation concerning the north). From the DSM, the arrangement of the roof segments was established using the "aspect" tool from the Spatial Analyst toolbox in ArcGIS. This tool applies an algorithm to determine the change in the downslope direction of a cell relative to its 8 neighbors [74]. Its execution provides a raster in which each cell contains a value in degrees that defines its illumination angle and is associated with one of the 9 orientation classes (flat and eight cardinal points).

**Fig. 6:**
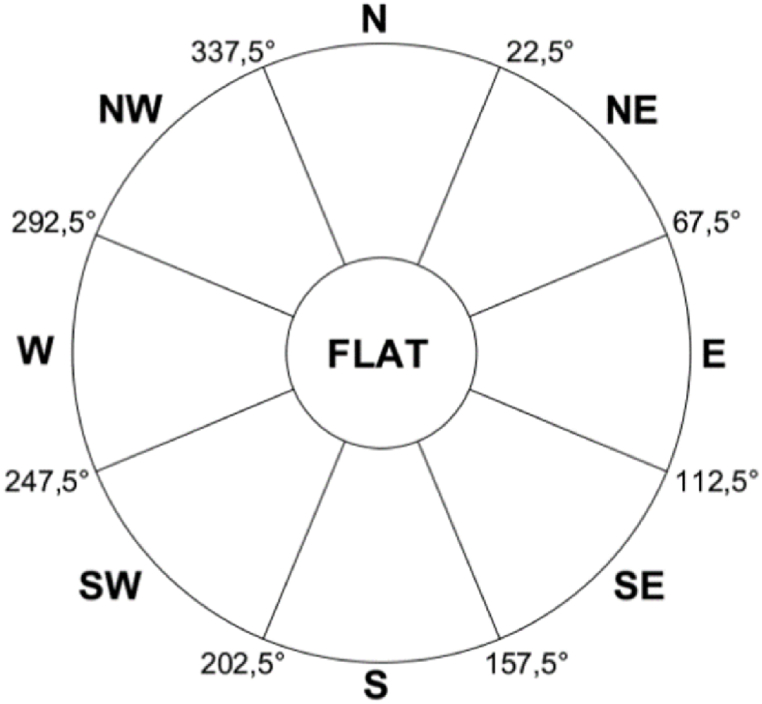
Roof segment orientation classes (adapted from Ref. [47]).

A majority filter is used to reduce orientation variations caused by LiDAR data noise [44]. To determine the orientation of each roof segment, the average angle of all cells within the roof footprint perimeter was calculated [54]. The result is a raster that shows the orientation of the cells, as shown in Fig. 5c and is classified according to the ranges in Fig. 6.

#### 4.2.3 Solar radiation

The amount of SR received by a roof is not only limited by the roof's own characteristics (slope and orientation). It is also influenced by shadows cast by external elements in the surrounding area, such as buildings or vegetation. The incident radiation was analyzed using the “area solar radiation” tool from the Spatial Analyst toolbox in ArcGIS. This tool calculates the amount of solar energy received by each cell of the DSM using the hemispherical viewshed algorithm developed by Fu and Rich [75,76]. The calculations take into account factors such as azimuthal directions, atmospheric transmissivity, and the proportion of diffuse radiation, but they do not consider the radiation reflected by adjacent surfaces [77].

This tool was parameterized to determine the insolation received throughout the DSM during one year. In this way, it calculates the incident radiation on the DSM roof cells while considering the reduction caused by nearby obstructing elements. The required values for the proportion of diffuse radiation and transmissivity were set at 0.3 and 0.4, respectively, to simulate the clear sky atmospheric conditions of the area [63]. With these values, annual solar radiation of approximately 1696 kWh/m^2^ was obtained in ArcGIS, a value reported in Ref. [78] based on measurements from a weather station near the analysis area. After execution, a raster is produced in which each cell contains the total radiation received during the year in Wh/m^2^, as shown in Fig. 5d.

#### 4.2.4 Shading

The “area solar radiation” tool in ArcGIS offers the option to generate a raster where each cell contains the value of hours of direct radiation it receives throughout the year (Fig. 5e). This image provides a visual reference of how shading affects roofs so that excessively shaded areas can be excluded during the implementation of PVs.

### 4.3 Roof analysis

To determine the roof segments suitable for PVs installation, the DSM cells are evaluated based on their attributes: slope, received radiation, and available area. The cells and roofs have not been segregated by their orientation since the study area is located at a latitude of −3.27° (close to the equator). Research such as [79,80] concludes that in equatorial regions, this factor does not significantly limit PVs performance as it does in higher latitudes. In this research, the cells belonging to roof segments are considered suitable after completing the selection process based on the following criteria.

#### 4.3.1 Slope

**Fig. 7:**
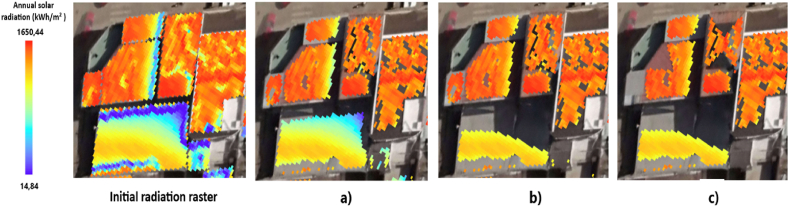
Segregation Process by: a) Initial radiation raster b) Slope c) Radiation d) Available Area.

The acceptable slope range for the roof cells in the slope raster was set between 0 and 30°. These values were referenced from the study [79] which concluded that PVs losses in Cuenca (latitude −2.9°, very close to that of Santa Isabel) become significant above 30° (see Fig. 7b).

#### 4.3.2 Incident radiation

The cells in the radiation raster must meet a minimum value of 1200 kWh/m^2^ of annual radiation, considering that the maximum value obtained in ArcGIS was 1650 kWh/m^2^. This limit was selected because the SR of non-ideal roofs (class 4 and 5) is below this value, as indicated by the statistics in section 5.1. By applying this segregation criterion and establishing a relationship between radiation values and annual hours of direct radiation, the raster generated with the Shading tool was analyzed. The results indicate that cells with 1650 kWh/m^2^ receive 4333 h of direct radiation, while cells with 1200 kWh/m^2^ receive 1923 h (see Fig. 7c).

#### 4.3.3 Available area

For a roof segment to be considered suitable, it must have an area greater than 10 m^2^. This value was set to allow for the installation of at least a small PVs, as typically, a free area of 7.43 m^2^ per installed kW is required [81]. Installation standards were also considered, which recommend that panels be placed at least 0.3 m from the roof edge [82] (see Fig. 7d). Applying these selection criteria allows for the creation of a map that visualizes the optimal areas of each segment for the installation of PVs. This ensures that these systems will have high efficiency and economic viability, making their future implementation more attractive. Some parameters are not considered as roofing accessibility, but buildings in the research area are lower than four floors, and most of them do have two floors, then it is anticipated that access for installation and maintenance will be relatively feasible.

## 5 Results

The processing and analysis of rooftops using ArcGIS allowed for determining the geometric characteristics of the roof segments and their surfaces suitable for PVs. These data are used to estimate, using Matlab, the theoretical, technical, and economic PVpot. The results of the latter will be used to calculate social and environmental benefit indicators for the analyzed neighbourhood.

### 5.1 Slope, orientation, and available area

**Fig. 8:**
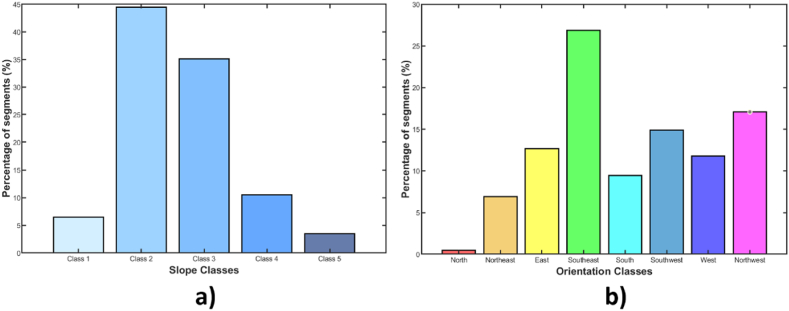
Percentage of roofs based on: a) slope b) orientation.

**Fig. 9:**
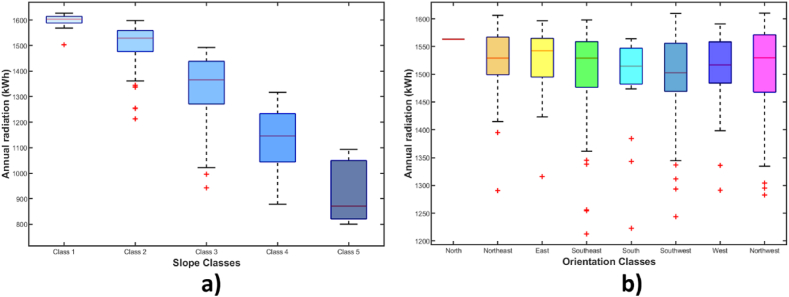
Box plots: a) relationship between radiation and slope for southeast-facing roofs, b) relationship between radiation and orientation for roofs with class 2 slope.

To improve the visualization of the results, in Fig. 8, Fig. 9, the slope and orientation classes follow the same color classification scheme as in ArcGIS presented in Fig. 5a and b. By determining the topographic characteristics of the 868 roof segments, it can be discerned that 86 % were grouped by slope into classes 1, 2 and 3 (0–30°) as shown in Fig. 8a. This majority represents areas with optimal inclinations according to Ref. [79] if the PV are installed with the same slope as the roofs. For panels to be installed on class 1 roofs, the PVs should be tilted to reduce dirt accumulation, allowing for natural cleaning by rain, as local rainfall has been shown to maintain PVs with dirt-related losses of less than 4 % [68,83].

The results of the roof classification by orientation are shown in Fig. 8b. It is observed that 26.8 % of the roof segments are oriented to the southeast. Considering that in areas near Cuenca-Ecuador, the performance of panels oriented to the east is slightly higher [79,85] In previous research conducted in the Andean equatorial climate, it has been established that the orientation of PVs minimally reduces overall energy production when the panels are set at an angle close to horizontal, with a maximum 7 % energy production reduction as consequence of orientation [86].

Fig. 9 presents box plots, which are useful for representing descriptive statistics of the data under analysis. The central red line reflects the median of the data set, the coloured boxes represent the 25th and 75th percentiles, the minimum and maximum values are indicated by the dashed lines, and outliers are represented by a cross symbol.

The relationship between incident radiation and roof slope is shown in Fig. 9a, for which 233 southeast-facing roof segments were analyzed. The results of this diagram confirm that radiation capture is higher on roofs with slopes closer to horizontal. It is observed that the incident radiation statistics are much lower starting from class 4 and 5 roofs, and the outliers in each class are caused by shading effects.

Fig. 9b shows the radiation-orientation relationship, which was obtained by examining the 386 roof segments with class 2 slopes. The statistics of radiation received by roofs with different orientations do not show significant variation, confirming that in equatorial zones, this is not a factor that significantly affects PV production. The highest radiation capture occurs on east-facing roofs, mainly due to lower cloud cover in the mornings (excluding the single north-facing roof). It is noted that the outliers are once again due to roofs affected by shading.

At the start of the roof segment segregation process, the total available area of these roofs was 43,842 m^2^. After separating the cells with a slope greater than 30°, the area was reduced to 38,528 m^2^. By applying the condition that the cells must receive at least 1200 kWh/m^2^ per year, the surface area was further reduced to 31,213 m^2^. Applying the condition that segments must exceed 10 m^2^ of suitable surface area, the useable area was set at 30,163 m^2^, representing 68.8 % of the total. Additionally, it was determined that out of the total 868 roof segments, 660 are suitable for implementing PV, corresponding to 76 % of the total.

### 5.2 Theoretical photovoltaic potential

The theoretical PVpot is determined based on the amount of incident irradiation on a surface, without considering the installation of a PV or its specific performance. Equation (1) was applied to calculate it [9]. Each roof segment is identified with i, their areas in square meters are assigned as Aseg, while SR annual represents their average annual solar radiation in kWh/m^2^.

(1) $${PotFV}_{theorical}=\sum_{i}{Aseg}_{i}∙{SRannual}_{i}$$

It was determined that the total available roof area in the study area is 43,842 m^2^. This allowed for the estimation of an annual theoretical PVpot of 62.39 GWh.

### 5.3 Technical photovoltaic potential

To determine the technical PVpot of the roofs in the area, the performance of the PVs and the reduction in useable area due to construction factors are considered. For this, a reduction factor technique based on the methodology proposed by Romero et al. [46] was used. This was applied to each segment to determine its useable area using Equation (2), while the technical PVpot was calculated using Equation (3). Table 3 summarizes the values assigned to the aforementioned factors.

(2) $$A_{USE}=C_{CON}∙C_{PRO}∙C_{SH}∙C_{AM}∙C_{RO}∙C_{RS}∙C_{SP}∙A_{AR}$$

Where.

• *A*_*USE*_: useable surface area in m^2^ for implementing PVs.
• *C*_*CON*_: reduction factor due to construction restrictions.
• *C*_*PRO*_: factor considering the number of historical or protected buildings.
• *C*_*SH*_: restriction due to mutual shading between buildings.
• *C*_*AM*_: restriction considering the space needed for access and maintenance.
• *C*_*RO*_: factor determined by the roof orientation.
• *C*_*RS*_: factor determined by the roof slope.
• *C*_*SP*_: restriction considering the spacing between panels.
• *A*_*AR*_: available area in m^2^ for each roof segment.

(3) $${PVpot}_{TEC}={SR}_{ANN}∙A_{USE}∙\eta_{EF}∙\eta_{TH}∙\eta_{AZ}∙\eta_{\mathrm{PR}}$$

Where.

• *PVpot*_*TEC*_: Technical photovoltaic potential.
• *SR*_*ANN*_: Annual solar radiation (in kWh/m^2^).
• *A*_*USE*_: Useable surface area (in m^2^) for implementing PV.
• *η*_*EF*_: Efficiency factor of the photovoltaic system.
• *η*_*TH*_: Efficiency factor related to the roof slope.
• *η*_*OR*_: Efficiency factor related to roof orientation.
• *η*_*PR*_: Performance ratio, considering real-world losses like temperature, shading, etc.

**Table 3:** Assigned values for reduction factors.

Factor	Value	Source	Justification
$C_{CON}$	0.8: Class 1 roof surface	[46]	
	0.9: Class 2 and 3 Class 1 roof surfaces		
$C_{PRO}$	1		No historic buildings are registered in cadastral data.
$C_{SH}$	1		Considered in the analysis performed with the ArcGIS solar radiation tool.
$C_{AM}$	0.97: Class 1 roof surface	[46]	
	1: Class 2 and 3 Class roof surfaces		
$C_{RO}$	1		Roofs are not segregated by their orientation.
$C_{RS}$	1		Considered in the analysis performed with the ArcGIS slope tool.
$C_{SP}$	0.62: Class 1 roof surface	[87]	
	1: Class 2 and 3 roof surfaces		
$\eta_{EF}$	0.16: PV crystalline silicon	[46]	
	0.11: PV thin film		
$\eta_{RS}$	0.9	[88]	
$\eta_{RO}$	1		Considered in the analysis performed with the ArcGIS solar radiation tool.
$\eta_{\mathrm{PR}}$	0.84	[88]	

To determine the percentage of demand that can be covered by PVs, the average annual electricity consumption of the 322 buildings within the analysis area was analyzed. This data was provided by the utility company Centrosur and corresponds to the year 2022. An annual consumption of 0.97 GWh was recorded. The technical PVpot with silicon panels (4.85 GWh) can supply 4.97 times the demand of the analyzed area; if thin-film panels (3.34 GWh) are considered, this value would be reduced to 3.42. The proposed procedure allowed for the selection of 119 roof segments with the highest PVpot to fully meet the demand, representing an area of 5877 m^2^ (13.4 % of the total).

To determine the number of panels that can be integrated into the useable area, the characteristics of panels available in the Ecuadorian commercial sector were examined. It was established that a 550 Wp monocrystalline solar panel (one of the most commercialized) occupies an approximate area of 2.6 m^2^. Considering this data, the number of panels that can be installed in the useable area of each roof segment was calculated, with a total of 9754 panels of the mentioned model that could be installed.

### 5.4 Economic photovoltaic potential

To evaluate the economic PVpot, a possible scenario for the implementation of photovoltaic roofs is proposed. The demand of the analyzed neighbourhood would be met by installing PVs with a total peak power of 6968.92 kW, considering a capacity factor of 16 % (1400 h). The LCOE of this project is determined by applying equation (4) [9].

(4) $$LCOE=\frac{\sum_{t}\left({CAPEX}_{t}+{OPEX}_{t}\right)∙{\left(1+d\right)}^{-t}}{\sum_{t}\left({PVout}_{t}\right)∙{\left(1+d\right)}^{-t}}$$

For the calculations, a value of 25 is assigned to *t*, since maintenance costs may exceed the economic benefits of continuing operation beyond this period [89] and most PV systems have warranties that guarantee at least 80 % efficiency during that time [90]. The *CAPEXt* represents the capital expenditures, which was determined considering an average price of $1150 per installed kW and an interest rate of 8 %. The operation and maintenance costs are established in *OPEXt* with an initial value of $26/kW [91] and an annual increase of 2 %. For the energy production *PVoutt*, an annual degradation of 0.5 % of the panels was considered. The factor *d* represents the discount rate, which was assigned a value of 10 % according to Ref. [20].

The obtained LCOE is 12.37 c$/kWh, a value that aligns with the results of previous research [92]. In that study, the LCOE of PVs was determined in a realistic context across four of the most populated cities in Ecuador. Additionally, the calculated LCOE shows a 32.4 % decrease compared to the values obtained in 2017 in the study [91]. These results are consistent with data from reports such as [9] or [20], which highlight the continuous reduction of this value for PVs. If this trend continues, the LCOE shortly could match or even be lower than, for example, the 9.2 c$/kWh corresponding to the cost of electricity for the residential category [93]. The current utility electricity price is lower than the retail price of the power from the utility since the electricity is subsidized by the government, so payback time for PV integration to buildings is not achieved. But if we consider the real power prices, estimated for the year 2022 in about 15,6 c$/KWh [94], the payback time could be reached, and it is dependent of the size of each solar system accordingly to its power capacity.

### 5.5 Environmental benefit

This section analyzes the environmental benefits of implementing the energy generation system described in the previous section. For this purpose, the emissions of tons of CO₂ avoided have been calculated by applying formula 5.

(5) $$Emissions=EF∙{PVout}_{t}$$

The emission factor (EF), expressed in tons of CO₂/MWh, allows the calculation of CO₂ emission reductions in the process of electricity generation using renewable sources. Meanwhile, PVoutt represents the energy production during the project's useful life. In the calculation, an EF value of 0.2957 tons of CO₂/MWh was considered, specific to a solar generation project [95]. It was determined that the energy produced over 25 years, considering the degradation of the panels, is 23 GWh. By implementing the mentioned project, 6805 tons of CO₂ emissions could be avoided in the environment over the project's lifetime.

### 5.6 Social benefit

The jobs required to install and operate the photovoltaic project are determined using equation (6) [91]. The number of jobs (job-years/GWh) is calculated by considering the years of operation (t), the project's annual operating hours (T), the jobs needed for construction, installation, and maintenance (CIM), and the personnel required for operation and maintenance tasks (OM).

(6) $${jobs}_{tot}=\left(\frac{CIM}{t}+OM\right)∙\frac{1000}{T}$$

For the calculations, the following values were assigned: *t* = 25 years, *T* = 1400 h, *CIM* = 19.7, *OM* = 0.7. The last two values were taken from the report [96]. The job creation factor was established at 1.06 job-years/GWh. This means that during the implementation and useful life of the project, 24.4 jobs can be created, which is significant considering that the analyzed area is 0.1 km^2^.

Table 4 presents a summary of the results obtained during the development of this research.

**Table 4:** Summary of results.

Parameter	Value	Unit
Analyzed Area	0.1	km^2^
Available Roof Area	43,842	m^2^
Theoretical PVpot	62.39	GWh
Area Suitable for PV	30,163	m^2^
Suitable Roof Segments	76	%
Technical PV Power with Silicon Panels	4.85	GWh
Technical PV Power with Thin-Film Panels	3.34	GWh
Demand in the Analyzed Area	0.97	GWh
Area Required to Meet Demand with Silicon Panels	5877	m^2^
Power with PV to Meet Demand	6968.92	kWp
LCOE	12.37	c$/kWh
Emissions Avoided by Implementing Solar Project	6805	Tons
Jobs Created by Implementing Solar Project	24.4	Jobs-year

## 6 Discussion and future work

This study aimed to establish a replicable LiDAR methodology for determining the PVpot in urban environments. The characterisation of roof area, slope, and orientation using this method greatly exceeds the accuracy of methodologies such as [56] or [58] which simplify roof shapes. The time required for this characterisation is significantly reduced compared to studies like [29,30] or [57], which necessitate on-site inspection procedures.

The annual theoretical PVpot of the roofs in the analyzed area (0.1 km^2^) is 62.39 GWh. This result nearly doubles the 34 GWh value presented by Redweik et al. [35], whose study examined roofs in a slightly larger area (0.16 km^2^). The difference in results is because, at higher latitudes, the SR captured by roofs over the year is lower due to seasonality. Additionally, for these locations, many roofs are excluded from the technical PVpot assessment process due to their less favourable orientation for PV implementation, which significantly reduces the useable implementation areas.

The estimated technical PVpot in this study can supply 4.97 times the value of the electricity demand if silicon panels are used. This result contrasts with similar studies such as [46] or [47], which indicate that the PVpot would only cover 77 % and 38 % of the demand, respectively. This situation is evident, for instance, in Ref. [51], where 56 % of the roofs are suitable for PV, or in Ref. [44], where it is shown that 48.6 % of the total roof area meets this condition. These values are exceeded by the results of this study, as the analyzed area is located near the equator. This location benefits from a 76 % suitability rate for roof segments and 68.8 % for the total area for PV, as no orientation segregation process is applied, and the received RS shows minimal variation throughout the year.

Compared to studies conducted in Ecuadorian cities, differences have been found with work such as that of Tian et al. [56], which determines that 21 % of the roof area has sufficient PVpot to meet demand. Our results indicate that only 13.4 % is required. In the case of the study by Barragán et al. [58], it is estimated that energy production with photovoltaic roofs can exceed demand by 3.19 times, while our calculations show this value reaches 4.97, possibly due to the lower population density.

When comparing the proposed methodology with the work developed by Bayrakci et al. [44], Gagnon et al. [54], or Mansouri et al. [47], similarities are noted in the process of selecting suitable roof surfaces for PV. However, this study has applied construction reduction factors to suitable areas, allowing for a more accurate approximation of a real implementation scenario. Additionally, a Matlab script has been incorporated to further complement and automate the PVpot calculation process.

The results of the PVpot calculation with the proposed method can be improved by considering the non-linear efficiency of PV panels, as seen in Ref. [45], or by using SR measurements, as in Ref. [47]. This would allow for better modelling and approximation of this factor using analysis software such as ArcGIS.

The layer was manually created to achieve accurate results in this study since the analysis area is relatively small. However, the methodology described applies to larger contexts (provincial or national) by utilising LiDAR data with building footprint layers created using deep learning techniques, available in Ref. [97] or [98]. These data would eliminate the need for an orthophoto to define footprints and would save time in creating the mentioned layer.

**Fig. 10:**
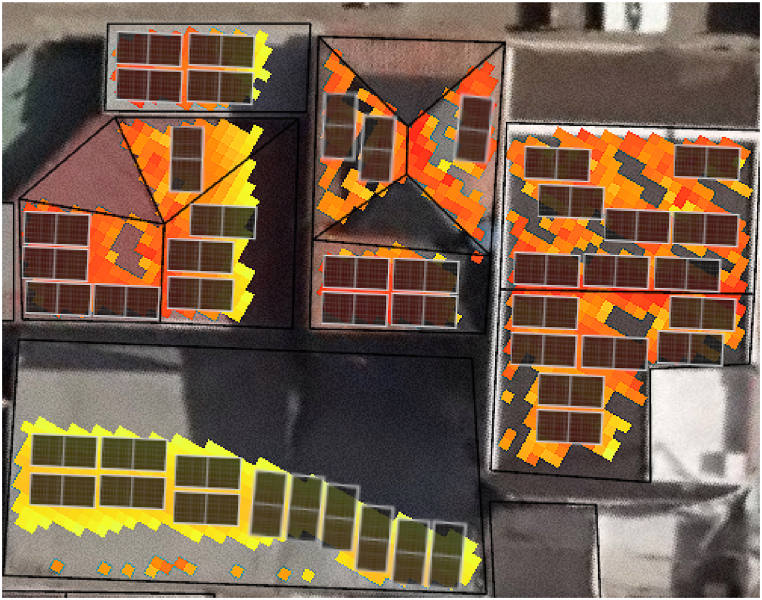
Manual distribution of PVs on suitable surfaces.

The suitable surface area for installing PVs on each roof segment was determined. For a real implementation, it is necessary to optimally place the PVs on suitable surfaces. Fig. 10 illustrates this situation using a manual process. For large areas, this approach is not efficient due to its high time demand. To address this issue, an algorithm for fitting PVs on roofs similar to the one proposed in Ref. [49] can be developed. There is an opportunity to improve this algorithm, as it typically considers the entire roof surface for PV placement without segregating less suitable areas. By applying the methodology of this research, a raster of suitable surfaces can be obtained, allowing the algorithm to optimally place PVs based on the aforementioned raster.

In Fig. 10, the manual placement of panels on suitable roof surfaces is illustrated. The process involves determining the areas where PV panels can be installed effectively, considering factors such as roof orientation and available surface area. This manual approach is demonstrated visually to show how PV panels are distributed across the identified suitable surfaces. For extensive areas, manual distribution may not be efficient due to time constraints, highlighting the need for automated algorithms to optimise PV placement.

## 7 Conclusions

This article presented a LiDAR methodology for estimating the theoretical, technical, and economic PVpot of roofs in a typical urban center in Ecuador. It contributed to the development of a Matlab script for analysing roof statistics and calculating PVpot using reduction factors, regardless of the size of the analyzed area. The potential of LiDAR technology was demonstrated in accurately characterising roof geometries. This information serves as the foundation for estimating PVpot and extracting optimal surfaces from each roof. The results show that between 4.85 GWh and 3.34 GWh can be generated annually, depending on the technology, which can meet up to 4.97 times the consumption. Compared to reference cases, this highlights the unique advantages of the Andean Ecuadorian climate.

The mentioned advantages, along with potential future improvements, could establish the method presented in this research as a standard for estimating PVpot in urban environments, facilitating the expansion of solar rooftops in Ecuador, where the determination of this value is still in an early stage. This hypothesis is supported by the fact that the current methodology could already generate maps of suitable areas for PV installation for each roof segment. This directly benefits potential users who wish to install PVs for self-consumption, eliminating inspection costs to establish these zones.

A current barrier to the use of LiDAR in Ecuador is the low or non-existent availability of LiDAR point clouds for extensive urban areas. Local governments or public and private electricity sector entities could increase their interest in using this technology to determine the PVpot of cities by reviewing the presented methodology. We hope that the results of this article will encourage favourable decisions for adopting photovoltaic roofs. The continuous decrease in the levelized cost of electricity (LCOE) for these systems, combined with reduced losses from distribution and transmission, makes them an attractive option for electrifying cities. Additionally, PV systems can contribute to the energy matrix transformation in Ecuador by diversifying and reducing reliance on conventional generation technologies. The massive integration of PV systems on rooftops also requires an analysis of the implications for the grids to accommodate multiple smaller systems generating surpluses. This demands that the grids be configured to have the capacity to manage bidirectional energy flows, which is necessary work for the future.

## CRediT authorship contribution statement

**Andrés Idrovo-Macancela:** Writing – original draft, Visualization, Validation, Methodology, Investigation, Formal analysis, Data curation, Conceptualization. **Marco Velecela-Zhindón:** Visualization, Validation, Methodology, Investigation, Formal analysis, Data curation, Conceptualization. **Antonio Barragán-Escandón:** Writing – original draft, Supervision, Resources, Investigation, Funding acquisition, Data curation, Conceptualization. **Esteban Zalamea-León:** Writing – review & editing, Writing – original draft, Resources, Funding acquisition, Formal analysis, Conceptualization. **Danilo Mejía-Coronel:** Writing – review & editing, Validation, Formal analysis.

## Data availability

Research-related Data is not stored in publicly available repositories, and Data will be made available on request.

## Ethics declarations

Review and/or approval by an ethics committee is not needed for this study because we don't work with humans or animals.

## Declaration of competing interest

The authors declare the following financial interests/personal relationships which may be considered as potential competing interests: Edgar Antonio Barragan Escandon reports financial support was provided by 10.13039/100016969Salesian Polytechnic University. Esteban Zalamea Leon reports administrative support and writing assistance were provided by 10.13039/501100018776University of Cuenca. Andres Homero Idrovo Macancela reports administrative support, statistical analysis, and writing assistance were provided by 10.13039/100016969Salesian Polytechnic University. Marco Vinicio Velecela Zhindon reports administrative support, statistical analysis, and travel were provided by 10.13039/100016969Salesian Polytechnic University. Danilo Mejia Coronel reports statistical analysis was provided by University of Cuenca. This document is part of a degree project for the attainment of the Master's degree from the Universidad Politécnica Salesiana. If there are other authors, they declare that they have no known competing financial interests or personal relationships that could have appeared to influence the work reported in this paper.
